# A comparison of the use of contrast media with different iodine concentrations for enhanced computed tomography

**DOI:** 10.3389/fphys.2023.1141135

**Published:** 2023-03-30

**Authors:** Yu Du, Ya-Ning Wang, Qi Wang, Xiao-Hui Qi, Gao-Feng Shi, Li-Tao Jia, Xiang-Ming Wang, Jia-Bao Shi, Feng-Yun Liu, Li-Jia Wang, Xiang Liu

**Affiliations:** Department of Radiology, The Fourth Hospital of Hebei Medical University, Shijiazhuang, China

**Keywords:** computer tomography, enhancement, iodine contrast medium, liver, diagnostic

## Abstract

**Objective:** In this study, we compared the enhancement of blood vessels and liver parenchyma on enhanced computed tomography (CT) of the upper abdomen with two concentrations of contrast media (400 and 300 mg I/mL) based on similar iodine delivery rate (IDR) of 0.88 and 0.9 g I/s and iodine load of 450 mg I/kg.

**Methods:** We randomly assigned 160 patients into two groups: iomeprol 400 mg I/mL (A group) and iohexol 300 mg I/mL (B group). The CT attenuation values of the main anatomical structures in the two groups with different scanning phases were measured and the image quality of the two groups was analyzed and compared. The peak pressure and local discomfort (including fever and pain) during contrast medium injection were recorded.

**Results:** The mean attenuation value of the abdominal aorta was 313.6 ± 29.6 in the A group and 322.4 ± 30.1 in the B group during the late arterial phase (*p* = 0.8). Meanwhile, the mean enhancement values of the portal vein were 176.2 ± 19.3 and 165.9 ± 24.5 in the A and B groups, respectively, during the portal venous phase (*p* = 0.6). The mean CT values of liver parenchyma were 117.1 ± 15.3 and 108.8 ± 18.7 in the A and B groups, respectively, during the portal venous phase (*p* = 0.9). There was no statistical difference in image quality, peak injection pressure (psi), and local discomfort between the two groups (*p* > 0.05).

**Conclusion:** When a similar IDR and the same iodine load are used, CT images with different concentrations of contrast media have the same subjective and objective quality, and can meet the diagnostic needs.

## 1 Introduction

When abdominal computed tomography (CT) examinations are performed, the contrast injection protocol should ensure that relatively stable contrast enhancement of vessels and parenchymal organs are obtained in different patients and examinations, while considering the following main parameters: contrast concentration, contrast volume, injection rate, injection time, and saline application, especially total iodine load and iodine delivery rate (IDR) (Bae, 2010). Recently, numerous studies have shown that IDR is an injection parameter that consistently maintains vascular image quality in different CT examinations, even when contrast media of different compositions and concentrations are utilized (Paparo et al., 2014). The IDR can be calculated according to the following formula: IDR = ([I]/1000) × FR, where [I] is the iodine concentration (mg I/mL) and FR is the injection rate of contrast medium (mL/s) (Rengo et al., 2012). In addition, the enhancement degree of the liver parenchyma is related to the total amount of iodine used per kg of body weight. The total amount of contrast media needs to be calculated based on the body weight of patients and the concentration of contrast media.

Moreover, the iodine concentration of 500–750 mg/kg of body weight is associated with good liver enhancement. Recent studies have confirmed the importance of IDR in CT angiography (Cho et al., 2015; Lubbers et al., 2018). However, only one previous study focused on comparing the application value of high- and low-concentration contrast media in dynamic-enhanced CT of the liver, including during late arterial, portal venous, and equilibrium phases. The enhancement effects of two different contrast media (320 mg I/mL iodixanol at an injection rate of 5 mL/s and 400 mg I/mL iomeprol at an injection rate of 4 mL/s) were compared under the fixed IDR of 1.6 g I/s and the total iodine load of 40 gI. In this study, however, the total iodine load per kilogram of body weight was not separately calculated for each patient (Rengo et al., 2012). The results of this study showed no significant difference in the degree of enhancement of vessels and tissues on the CT with these two contrast media.

Based on these results, we conjecture that the potential effects of differences in molecular structure can be disregarded for different concentrations of contrast media as the same enhancement effect is obtained. In addition, viscosity is determined by the molecular structure of the contrast medium (such as differences in molecular size and organic side chain composition), which is also influenced by osmotic pressure and temperature. Therefore, the difference in viscosity of molecules in contrast media with different concentrations is mainly related to osmotic pressure and temperature. In this study, we mainly aimed to compare the difference in the enhancement of aorta, portal vein, and liver parenchyma on CT with two concentrations of contrast media (400 and 300 mg I/mL) under the similar IDR (0.88 and 0.9 g I/s) and the same iodine load per kilogram of body weight (450 mg I/kg). Furthermore, we evaluated the effect of the concentration and viscosity of contrast media on the peak injection pressure.

## 2 Materials and methods

This study was a single-center, prospective, and randomized study comparing the enhancement effects of two concentrations of contrast media, whose protocol was approved by the Ethics Committee of our hospital. Patients signed an informed consent form before inclusion. In this study, 160 patients underwent dynamic-enhanced liver CT from February 2022 to March 2022. They were randomized into two groups (A and B), with a specific concentration and brand of contrast medium used for each group. Exclusion criteria of patients were as follows: patients with contraindications to routine CT examinations (that is, pregnant women); patients with contraindications to contrast medium use (including severe renal dysfunction, creatinine clearance rate less than 30 mL/min, use of a modification of diet in renal disease equation, severe cardiac dysfunction [New York Heart Association class III and IV], and a history of severe allergic reactions to iodine contrast media) (Lubbers et al., 2018). Patients with known cirrhosis were excluded because the parenchymal fibrosis in these patients could impair liver enhancement and reduces portal vein perfusion. The exclusion criteria after the completion of the examination were patients with motion artifacts and abnormal liver perfusion due to chemotherapy or other drug therapies. We excluded patients with relatively large primary tumors or secondary lesions covering the entire liver or multiple segments.

### 2.1 Image acquisition and contrast medium injection parameters

Scanning was conducted on SOMATOM Force (Siemens Medical Solutions, Forchheim, Germany) with the following specific parameters: automatic tube voltage (Care kV), automated attenuation-based tube current modulation (CARE Dose 4D, 147 Eff mAs), collimation (192 × 0.6 mm), pitch (0.6), rotation time (0.5 s), reconstruction layer thickness (1 mm), and layer spacing (1 mm). The images were routinely reconstructed with the ADMIRE algorithm.

The volume of iodine contrast media was calculated in milliliters and the weight of the patients was recorded and expressed in kilograms to ensure the same iodine load for patients in both groups. The volume of contrast media was calculated with the formula—the volume of contrast media (mL) = body weight (kg) × 1.125, for patients in the A group (400 mg I/mL), and the formula—the volume of contrast media (mL) = body weight (kg) × 1.5, for patients in the B group (300 mg I/mL). Based on previous usage experience, the total iodine load was set at 450 mg I/kg body weight, divided by the concentration of contrast media to obtain two parameters of 1.125 and 1.5.

The contrast media in both groups were pre-warmed to 37°C before use and injected into the antecubital vein with a double-barrel high-pressure syringe (Accutron CT-D, MEDTRON, GERMANY) *via* an intravenous indwelling needle (22G). Under similar IDR (0.88 and 0.9 g I/s), patients in the A group were injected with iomeprol (Patheon Italia S.P.A, 400 mg I/mL) at a rate of 2.2 mL/s and those in the B group were injected with iohexol (Shanghai Sitaili Company, 300 mg/mL) at a rate of 3 mL/s. After contrast medium injection, patients were injected with saline (50 mL) at the same injection rate (2.2 mL/s and 3 mL/s for the A and B groups, respectively) for flushing. The monitoring was conducted by placing the region of interest (ROI) within the aorta at the level of the diaphragm using the bolus-tracking technique to reduce the effect of differences in cardiac function, with the trigger threshold set at +100 Hu. The scanning was first performed in the cephalopodal direction and then the late arterial phase was scanned 15 s after reaching the threshold. The portal phase was scanned 35 s after completing the arterial phase, and the equilibrium phase was scanned 120 s later.

### 2.2 Subjective and objective evaluation of CT images

Quantitative and qualitative analyses of the CT images were performed jointly by two experienced abdominal radiologists blinded to the enhanced scanning protocol of each patient. The quantitative analysis was performed: the CT attenuation values of crucial anatomical structures (including the aorta, portal vein, and liver parenchyma) were measured and expressed as Hounsfield Units (HU); with the standard area of the circular ROI set at 1 cm^2^, the images of plain and enhanced scanning (that is, arterial and portal venous phases) were measured. When liver nodules were present, the ROI was carefully placed outside the lesion-associated perfusion abnormality area to avoid interference.

The CT attenuation value of each structure in each phase minus that of the plain scanning was recorded as the enhancement value in different enhancement phases and expressed as the mean CT attenuation value ± standard deviation (SD) to facilitate further statistical analysis. The CT attenuation values were measured in the center of the vessel at three different layers of the abdominal aorta (first, middle, and last layer). The CT attenuation values were measured in the main portal vein and within the left and right major branches of the liver. The CT attenuation values of the liver parenchyma were measured in six different regions, including the left lobe (segments II and III), the right lobe (segments VII and VIII), and below the hepatic hilar (segments V and VI), with large vessels, visible bile ducts, or other lesioned areas avoided. The attenuation values of the abdominal aorta were only measured in the arterial phase and those of the portal vein and liver parenchyma were measured in the portal venous phase. We measured the mean attenuation values of crucial anatomical structures (including the abdominal aorta, portal vein, and liver parenchyma). We assessed whether there was a significant difference between the A (400 mg I/mL) and B (300 mg I/mL) groups. We also conducted more detailed analysis results, including mean attenuation values for each part of the above anatomical structures (including the upper, middle, and lower abdominal aorta, the main, right and left branches of the portal vein, and hepatic segments of the right and left lobes of the liver).

Thirty days after the quantitative analysis, two reviewers jointly reviewed all CT images under blinding to the contrast medium injection protocol and assessed arterial phase enhancement quality (AEQ) and liver enhancement quality on portal phase (LEQ) with a semi-quantitative scale (1 = poor; 2 = good; 3 = excellent) (Paparo et al., 2014). The reviewer needs to evaluate whether the enhanced image is satisfactory for diagnostic purposes (that is, adequate contrast and resolution of the aorta, portal vein, or liver parenchyma and adjacent anatomic structures).

### 2.3 Injection pressure

An Accutron CT-D CT High-Pressure Injector (MEDTRON, Saarbrucken, Germany) was used to monitor the injection pressure curve. The peak injection pressures, expressed in pounds per square inch (psi), were automatically calculated and displayed on the control screen of the high-pressure injector. The injector was attached to a 22G intravenous indwelling needle, ensuring maximum standardization of injection conditions.

### 2.4 Local discomfort at the injection site

Local discomfort at the injection site (such as local pain and fever) was evaluated with a 4-point rating scale (0 = no; 1 = mild, tolerable; 2 = moderate; 3 = severe, intolerable). The adverse reactions of patients were monitored within 1 h after the completion of the CT examination, such as fever and metallic taste, which are common non-allergic adverse reactions to intravenous iodine contrast medium (Zhang et al., 2016).

### 2.5 Statistical analysis

In this study, the mean values of enhancement (HU) in the hepatic artery, portal vein, and liver were mainly between the two groups. Categorical variables are expressed as numbers and percentages, ordinal variables as the median (min-max) of the corresponding range, and continuous variables as mean ± standard deviation. The normal distribution of the data set was evaluated with the D'Agostino-Pearson test. The non-parametric test was used instead of the parametric test to analyze data without normal distribution. The significance of differences between the two groups was assessed with the independent samples *t*-test for continuous variables with normal distribution. In contrast, the independent samples were analyzed with the non-parametric Mann-Whitney test for non-normally distributed variables. Fisher’s exact probability test was used for categorical data. Two-sided *p* < 0.05 was considered statistically significant. All statistical analyses were performed with SPSS 21.0 statistical software.

## 3 Results

### 3.1 General information of patients in the two groups

A total of 160 patients were enrolled in this study, exceeding the minimum sample size. In the A group, there were 40 males and 40 females with a mean age of 61.5 ± 12.3 years, a mean weight of 60 ± 14.3 kg, and a mean body mass index (BMI) of 20.8 ± 4.2. The B group included 42 males and 38 females with a mean age of 62.8 ± 11.4 years, a mean weight of 60.7 ± 13.5 kg, and a mean BMI of 20.2 ± 3.8. Most patients (121/160, 75.6%) underwent dynamic-enhanced abdomen CT for staging/re-staging/follow-up of malignancy with suspected liver metastases. In contrast, the remaining patients (39/160, 24.4%) were referred for the feature of focal liver lesions. There was no significant difference in demographic and anthropometric characteristics between the two groups (*p* > 0.05).

### 3.2 Results of quantitative and qualitative analyses

On the plain scan, the CT attenuation value of the abdominal aorta was 45.2 ± 5.1 in the A group and 43.3 ± 4.6 in the B group, that of the portal vein was 44.7 ± 5.6 in the A group and 41.8 ± 4.1 in the B group, and that of liver parenchyma was 63.3 ± 4.5 in the A group and 64 ± 7.1 in the B group. No significant difference in the mean CT attenuation values was observed between the two groups (*p* > 0.05).

**FIGURE 1:**
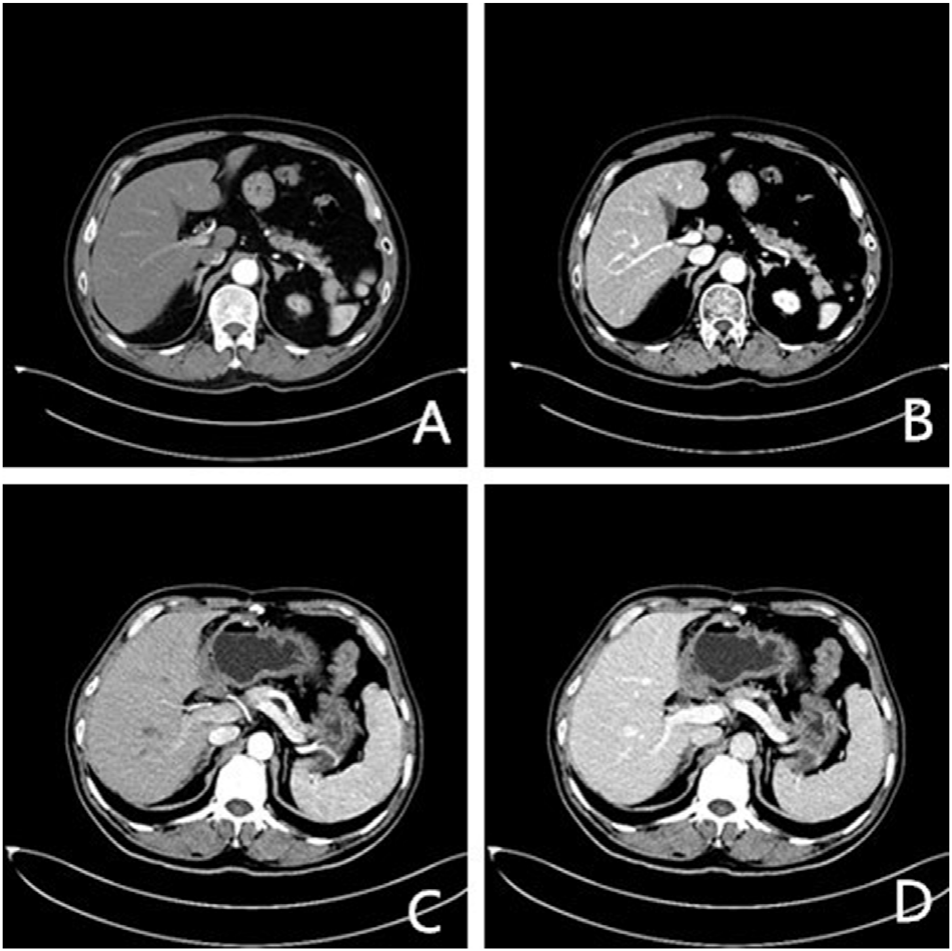
Axial enhanced CT images of two patients (arterial and venous phase). **(A)**, **(B)** a 56-year-old man with lung cancer after chemotherapy (400 mg I/ml); **(C)**, **(D)** a 64-year-old man after resection of gastric cancer (300 mg I/ml).

The mean attenuation values of the abdominal aorta during the late arterial phase were 313.6 ± 29.6 in the A group and 322.4 ± 30.1 in the B group (*p* = 0.8). During the portal venous phase, the mean enhancement values of the portal vein were 176.2 ± 19.3 and 165.9 ± 24.5 in the A and B groups, respectively (*p* = 0.6). The mean CT values of liver parenchyma were 117.1 ± 15.3 and 108.8 ± 18.7 in the A and B groups, respectively (*p* = 0.9). Table 1 summarizes the mean attenuation values of the main anatomical structures during different enhancement phases and the results of the quantitative analysis. Meanwhile, no significant differences were found between the two groups in terms of the mean CT attenuation values for each part of the crucial anatomical structures (including the upper, middle, and lower abdominal aorta, the main, right, and left branches of the portal vein, and hepatic segments of the right and left lobes of the liver) (Figure 1).

**TABLE 1:** Summary of the comparisons between the mean attenuation values of the key anatomical structures at different dynamic phase.

	Aorta (unenhanced phase)	*p*	Aorta (arterial phase)	*p*	Liver (unenhanced phase)	*p*	Liver (portal phase)	*p*	Portal (unenhanced phase)	*p*	Portal (portal phase)	*p*
Group A	45.2 ± 5.1	0.7	313.6 ± 29.6	0.8	63.3 ± 4.5	0.6	117.1 ± 15.3	0.9	44.7 ± 5.6	0.8	176.2 ± 19.3	0.6
Group B	43.3 ± 4.6		322.4 ± 30.1		64 ± 7.1		108.8 ± 18.7		41.8 ± 4.1		165.9 ± 24.5	

Qualitative analysis results (Table 2) revealed no significant difference in the AEQ and LEQ between the two groups (*p* > 0.05).

**TABLE 2:** Results of the comparison between two groups.

	Group A			Group B			*p* value
	Median	Min	Max	Median	Min	Max	
AEQ	3	1	3	3	1	3	0.60
LEQ	3	2	3	3	2	3	0.75

### 3.3 Peak injection pressure (psi)

**FIGURE 2:**
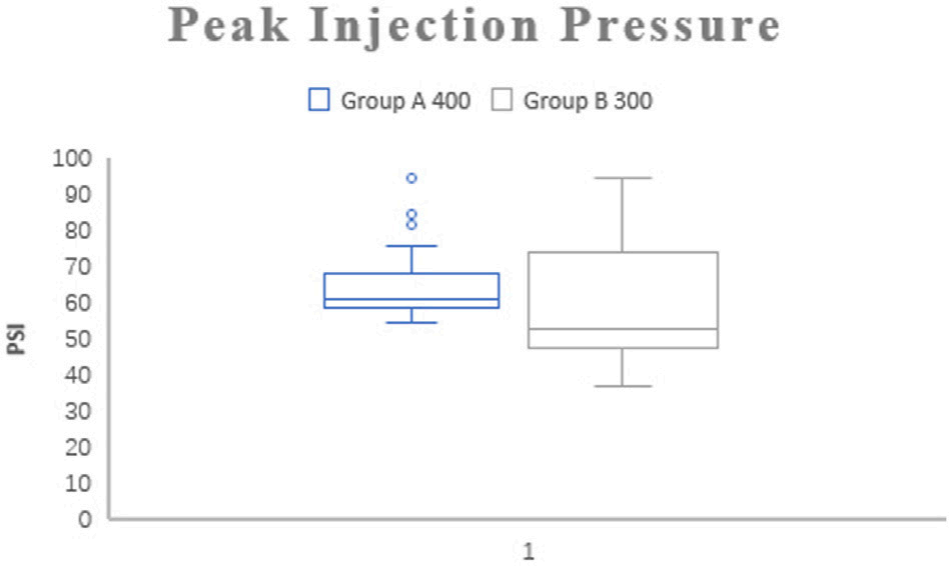
Bar graph representing median peak injection pressure between groups.

There was no significant difference in the A group (66, 59–95) compared to the B group (58, 47–98) (*p* > 0.05; Figure 2 and Table 3).

**TABLE 3:** Peak injection pressure (psi) of two groups.

	Group A	Group B	*p* value
psi	66, 59–95	58, 47–98	0.32

### 3.4 Local discomfort at the injection site

Local discomfort (pain and fever) at the injection site was not markedly different between the two groups, with a median score of 0 in both groups (*p* > 0.05). No cases suffered from local pain at the injection site in either group, while local warmth occurred in 8/80 (10%) patients in the A group, and 9/80 (11.3%) patients in the B group experienced local warmth (*p* > 0.05). There were no cases of extravasation and no mild, moderate, or severe allergic reactions to contrast media (Table 4).

**TABLE 4:** Local discomfort at the injection site of two groups.

	Group A	Group B	*p* value
Local discomfort (median score)	0	0	0.56
Local warmth	8/80 (10%)	9/80 (11.3%)	0.43
Extravasation	0	0	0.64
Allergic reactions	0	0	0.64

## 4 Discussion

Consensus on the injection protocols of iodine contrast media has long been lacking, which, to some extent, affects the credibility of the results of some of the previous studies that were designed to determine the iodine concentration needed to produce optimal vascular and parenchymal enhancement in CT examinations (Nakagawa et al., 2015; Jo et al., 2016). Previous studies elucidated that contrast media with higher iodine concentrations improved vascular and parenchymal organ enhancement better than contrast media with lower iodine concentrations (Shen et al., 2016; Ippolito et al., 2019). In addition, another study compared two different concentrations of contrast media from the same brand (iopamidol, 300 and 370 mg I/mL) under a fixed dose (100 mL) and injection rate (3 mL/s), which illustrated that the higher concentration of contrast media was associated with the higher enhancement values in the aorta, portal vein, and liver parenchyma (Kok et al., 2014). Nevertheless, the two main determinants of enhanced scanning (IDR and total iodine load) must be the same when the injection rate and volume of contrast media are adjusted appropriately according to the concentration. It allows for a more reliable comparison of the enhancement efficacy of different concentrations of contrast media.

Evidence suggests that IDR directly affects arterial vascular enhancement, while total iodine load influences the enhancement in the portal venous phase. In a recent study on enhanced positron emission, tomography/CT, a body surface area (22.26 g I/m^2^)-dependent contrast injection protocol with a fixed IDR of 1.29 g I/s was used to compare enhanced images obtained with two different iodine concentrations (iopromide, 300 mg I/ml vs. 370 mg I/ml) of contrast media from the same brand. In analyzing images obtained during the portal venous phase (70 s after the start of contrast medium injection, without the use of bolus tracking technique), in this study, we found no significant difference in the enhancement degree of all anatomical sites (ascending aorta, abdominal aorta, inferior vena cava, main portal vein, liver, kidney) in images obtained with these two concentrations of contrast media (Verburg et al., 2013). In our study, quantitative and qualitative analyses manifested no significant differences between the two groups of patients, thus confirming the finding of Verburg et al. regarding imaging of the portal venous phase and extending this finding to the late arterial phase. In the field of abdominal and liver imaging, Rengo et al. compared the mean attenuation values (expressed as mean contrast enhancement index) of crucial anatomical structures (abdominal aorta, portal vein, and liver parenchyma) under the use of two concentrations of two different contrast medium molecules (320 mg I/mL iodixanol and 400 mg I/mL iomeprol). They analyzed images at three phases (including late arterial, portal venous, and equilibrium phases). They found that both contrast media could exert similar enhancement effects at a fixed IDR of 1.6 g I/s. According to previous studies, IDR values of 1.2–1.6 g I/s ensure good tumor-liver contrast in blood-rich hepatocellular carcinoma, which is suitable for dynamic-enhanced CT scanning of the abdomen (Aschoff et al., 2017). The IDR used in our study was lower than that utilized by Rengo et al. (0.88 or 0.9 g I/s vs. 1.6 g I/s). The actual tube voltage was 90–100 kV due to the use of the automatic tube voltage technique in the scan, which elevated the CT attenuation values after the enhancement of the vessels and liver parenchyma. This result also confirms that the lower IDR combined with the automatic kV technique can provide enhanced CT image quality that meets clinical diagnostic needs. In addition, this injection protocol has the advantage of a lower injection rate, which is more conducive to patients with lower peripheral venous vascular quality.

As an IDR of 0.88 and 0.9 g I/s was used, the injection rate in this study was maintained reasonably low, with no contrast medium extravasation and a negligible incidence of local discomfort at the injection site in either group. In several previous studies, the peak injection pressures were recorded with a circulation phantom. In a prior study, a circulation phantom with physiological circulation parameters was used to compare four different concentrations (240, 300, 370, and 400 mg/mL) of contrast media (iopromide) (all pre-warmed at 37°C) under the fixed IDR (2.0 g/s) and the total amount of iodine (20 g). Through appropriate adjustment of the injection rate for different concentrations of contrast media, this study proposed that a fixed IDR ensured comparable mean CT attenuation values that did not correlate to the simulated vascular structures (ascending aorta, descending aorta, and coronary arteries) of the circulation phantom (Mihl et al., 2013). The authors of this study also evaluated the peak injection pressures of contrast media with different concentrations. They noted substantially higher peak injection pressures (expressed as psi) for the contrast media with higher iodine concentrations. In our study, there was no statistical difference in the median peak injection pressures between the A and B groups (*p* > 0.05), which may be due to the interaction of several factors. For instance, although the viscosity of contrast media varied after preheating (37°C), a lower injection rate was used for the high-viscosity contrast media in our study. The main limitation of the peak injection pressure analysis is that many patient-related factors and injection parameters may affect these measurement results. Unfortunately, little is known about such hydrodynamic parameters. Moreover, the injection conditions were fixed for different patients, especially using 22G intravenous indwelling needles and connecting tubes. No significant increase was observed in the incidence of local discomfort (pain and fever) at the injection site in the A group, in which the used contrast media had a higher osmotic pressure when compared to the B group. Our results indicate that appropriate IDR could be achieved even if the lower concentrations of contrast media have lower viscosity and osmotic pressure than the higher concentration of contrast media. It is speculated that these chemical and rheological properties of contrast media (especially viscosity) may be associated with numerous toxic side effects, including nephrotoxicity. The osmotic pressure of contrast media increases linearly, but their viscosity increases exponentially with molar concentration (European Society of Urogenital Radiology, 2019; ACR Committee on Drugs and Contrast Media, 2020). From the renal artery to the medulla, the contrast-containing body fluid in renal tubules gets gradually concentrated, accompanied by progressively elevated osmotic pressure. In contrast, the viscosity of the tubular fluid is enhanced exponentially. The high viscosity prolongs the intrarenal retention of contrast media, resulting in prolonged exposure of renal tubular epithelial cells to iodine contrast media and thus, causing nephrotoxic effects. Likewise, medullary hypoxia is another pathophysiological mechanism that causes oxidative stress and cellular damage. Iodine contrast media directly decreases renal medullary blood flow by enhancing plasma viscosity. In addition, viscosity-related contrast medium retention impairs renal tubular flow and elevates tubular pressure, further contributing to renal medullary hemodynamic damage. Consequently, contrast media with a higher viscosity may reduce renal medullary blood flow and glomerular filtration rates, thereby increasing the risk of nephrotoxicity. The adverse effects of high-viscosity contrast media may be more pronounced in dehydrated patients and the use of contrast media with low iodine concentrations may reduce the risk of nephropathy (Moss et al., 2017; Richards et al., 2018). A lower IDR (0.88 and 0.9 g I/s) can be ensured in routine clinical practice under the same iodine load (450 mg I/kg) by rapidly calculating the contrast volume and correctly setting injection parameters.

The following limitations exist in this study. Firstly, we only compared the enhancement effects of two concentrations of iodine contrast media. Therefore, further studies are needed to investigate the enhancement effects of other concentrations and brands of contrast media. Secondly, the study population was mainly made up of cancer patients with relatively low BMI. Accordingly, there may be limitations in extending the results of this study to other populations. A specific CT scanner and reconstruction algorithm were used in this study, and the results obtained when different types of CT scanners are used may differ.

## 5 Conclusion

With similar IDR and fixed total iodine load, different concentrations of contrast agents can exert the same effect on vascular and hepatic parenchymal enhancement, which can meet clinical diagnostic needs.

## Data Availability

The original contributions presented in the study are included in the article/supplementary material, further inquiries can be directed to the corresponding author.
